# PREPUBERTAL GYNECOMASTIA: A RARE MANIFESTATION OF MYOTONIC DYSTROPHY TYPE 1

**DOI:** 10.1590/1984-0462/2020/38/2018294

**Published:** 2020-02-14

**Authors:** Patrícia Sofia Ferreira Miranda, Ester Preciosa Maio Nunes Pereira, Joana Serra Caetano Baltazar Barreto, Margarida Maria Videira Henriques, Maria Alice Santos Cordeiro Mirante, Lina Maria Jesus Ferreira Cardoso Ramos

**Affiliations:** a Centro Hospitalar de Leiria, Portugal.; b Centro Hospitalar e Universitário de Coimbra, Portugal.

**Keywords:** Gynecomastia, Myotonic dystrophy, Steinert disease, Aromatase, Adolescent, Ginecomastia, Distrofia miotônica, Doença de Steinert, Aromatase, Adolescente.

## Abstract

**Objective:**

To present a case of bilateral gynecomastia in a prepubertal boy with autism spectrum disorder, diagnosed with myotonic dystrophy type 1.

**Case description:**

A 12-year-old boy with autism spectrum disorder presented at a follow-up visit with bilateral breast growth. There was a family history of gynecomastia, cataracts at a young age, puberty delay, and myotonic dystrophy type 1. The physical examination showed that he had bilateral gynecomastia with external genitalia Tanner stage 1. Neurologic examination was regular, without demonstrable myotonia. The analytical study revealed increased estradiol levels and estradiol/testosterone ratio. After excluding endocrine diseases, the molecular study of the dystrophia myotonica protein kinase gene confirmed the diagnosis of myotonic dystrophy type 1.

**Comments:**

A diagnosis of prepubertal gynecomastia should include an investigation for possible underlying diseases. This case report highlights the importance of considering the diagnosis of myotonic dystrophy type 1 in the presence of endocrine and neurodevelopmental manifestations.

## RESUMO

**Objetivo:**

Apresentar o caso de um adolescente pré-púbere com ginecomastia bilateral e transtorno do espectro autista, diagnosticado com distrofia miotônica tipo 1.

**Descrição do caso:**

Adolescente do sexo masculino de 12 anos, com transtorno do espectro autista, observado em consulta de seguimento por crescimento mamário bilateral. O paciente tinha antecedentes familiares de ginecomastia, catarata em idade jovem, atraso pubertário e distrofia miotônica tipo 1. À observação física, apresentava ginecomastia bilateral estádio 1 de Tanner. O exame neurológico era normal, sem miotonia aparente. O estudo analítico mostrou níveis elevados de estradiol e da relação estradiol/testosterona. Após exclusão de causas endócrinas, o estudo molecular do gene DMPK confirmou o diagnóstico de distrofia miotônica tipo 1.

**Comentários:**

Perante um quadro de ginecomastia pré-púbere, deve-se excluir doenças subjacentes. Este caso reforça a importância de considerar o diagnóstico de distrofia miotônica tipo 1 na presença de manifestações endócrinas e do neurodesenvolvimento.

## INTRODUCTION

True gynecomastia is diagnosed by the presence of a palpable fibroglandular mass that measures at least 0.5 cm in diameter and is located concentrically beneath the nipple-areolar complex.^1^ It is highly prevalent and often benign during the neonatal period, puberty, and in males over 50 years-old.^2^ ^,^ ^3^ ^,^ ^4^

Pubertal gynecomastia is a common and usually physiological disease, and spontaneous regression occurs within one to three years.^1^ ^,^ ^5^ The prevalence of pubertal gynecomastia ranges from 3.9 to 64.6% and, typically, it appears at least six months after the onset of male secondary sex characteristics, with the peak of incidence at Tanner stages 3-4 and testicular volume of 5 to 10 mL.^1^ ^,^ ^5^

On the other hand, prepubertal gynecomastia is characterized by the presence of breast tissue without other secondary sexual characteristics. It is rare and comprises 5% of gynecomastia referrals.^1^ ^,^ ^4^ Generally, it is considered a pathological sign of a possible endocrinopathy.^4^ ^,^ ^6^

Pathological gynecomastia is thought to be caused by the imbalance between estrogen related to androgen action at the breast tissue.^7^ Elevated serum estrogen levels may be the result of endogenous abnormal production, such as neoplasms secreting estrogen or precursors (e.g. Leydig or Sertoli cell tumors, hCG-producing tumors, and adrenocortical tumors), exogenous administration or, more commonly, increased extragonadal conversion of androgens to estrogens through tissue aromatase.^7^ Inversely, imbalance can also be caused by a decrease in serum androgen levels seen in primary (e.g. Klinefelter’s syndrome) or secondary hypogonadism, impaired androgen biosynthesis caused by enzymatic deficiency or medications and androgen receptor malfunction.^5^

Several medical conditions may be associated with pathological gynecomastia, such as hyperprolactinemia, hyperthyroidism, chronic diseases leading to malnutrition (e.g. cystic fibrosis, ulcerative colitis, liver disease, chronic renal failure, acquired immunodeficiency syndromes),^5^ and myotonic dystrophy type 1.

The authors present a case report of bilateral gynecomastia in a prepubertal boy with autism spectrum disorder. Molecular study of the dystrophia myotonica protein kinase (DMPK) gene confirmed the diagnosis of myotonic dystrophy type 1.

## CASE DESCRIPTION

A 12-year-old boy with autism spectrum disorder presented at a surveillance consult with bilateral breast growth, which was noticed on the last few months. He did not mention other symptoms, such as headache, vomiting, and polyuria polydipsia. Growth was unremarkable, with height on the 75^th^ centile and weight on the 75-90^th^ centile. His mother denied he had been exposed to medications, herbal medicine drugs, or estrogen-containing creams.

**Figure 1:**
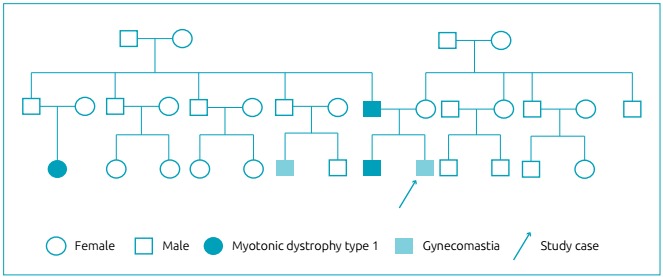
Genogram.

The family history showed that his parents were non-consanguineous. He had a first-degree cousin from his father’s family with gynecomastia since adolescence and other first-degree cousin from his father’s family with a recent diagnosis of myotonic dystrophy type 1. His father had cataracts diagnosed at 49 years-old and a history of puberty delay. The cousin’s diagnosis led to family genetic study, and his father and older brother were waiting for their results. His mother was healthy (Figure 1).

**Figure 2:**
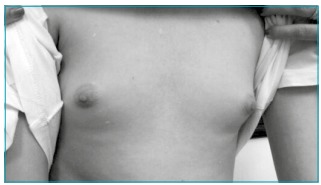
Gynecomastia (Tanner stage 3).

On physical examination, he had an eunuchoid phenotype and bilateral gynecomastia (Figure 2). Testicular volume was 2 mL bilaterally, with no palpable nodules, penis length was 5 cm (-2 standard deviation according to age), and there were sparse long, slightly pigmented curled pubic hairs at the base of his penis (external genitalia at Tanner stage 1 and pubic hair at Tanner stage 2). There was no tachycardia or tremor and nonpalpable thyroid. Body mass index was 18.8 kg/m^2^ (50^th^ to 75^th^ centile). There were no other relevant findings. Neurologic examination was regular, without demonstrable myotonia.

Analytical evaluation showed total testosterone=0.13 ng/mL (reference value=0.24±0.01 for male and Tanner stage 1), estradiol=49 pg/mL (reference value=4.6±5.8), estradiol/testosterone ratio=400 (reference value <10), LH=0.19 UI/L, and FSH 1.02=UI/L, prolactin=8.6 ng/mL (reference value=2.6-13.1), b-hCG=0.3 mUI/mL (reference value=0-3), TSH=2.33 uUI/mL (reference value=2.0±1.8), and free thyroxine=13 pmol/L (reference value=16.6±3.6). Complete blood count, kidney function, liver profile, and muscle enzyme levels were normal.

The presence of autism spectrum disorder in association with gynecomastia caused the consideration of genetic diseases. Karyotype was 46, XY normal and molecular genetic testing of FMR1 gene excluded X-Fragile syndrome. Molecular genetic testing of DMPK gene confirmed the diagnosis of myotonic dystrophy type 1. His father and brother were also diagnosed with the same condition.

Pubertal development was observed, and gynecomastia remained stable during the follow-up. On the last consult, at 14 years-old, he presented bilateral gynecomastia classified as Tanner stage 3, testicular volume of 8 mL on the left, and 6 mL on the right and pubic hair at Tanner stage 4. Analytical evaluation showed: total testosterone of 1.82 ng/mL (reference value=0.68±0.02 for male and Tanner stage 2), estradiol of 15 pg/mL (reference value=7.3±3.7), LH=3.7 UI/L, and FSH=6.9 UI/L. Electromyography was regular.

## DISCUSSION

Prepubertal gynecomastia, especially if unilateral, is extremely rare^3^ and should prompt an immediate evaluation for pathological causes and possible endocrine disorders.^1^ ^,^ ^4^

In our case report, gynecomastia occurred in a 12-year-old boy with external genitalia at Tanner stage 1 (testicular volume of 2 mL bilaterally) and, therefore, prepubertal. After exclusion of exogenous estrogen and drug intake by clinical history, we focused on possible endocrine and systemic non-endocrine diseases, especially those that could be part of a syndrome that occurs with autism spectrum disorder.

The analytical study revealed an increase in estradiol levels and in estradiol/testosterone ratio. Tumoral abnormal production of estradiol was unlikely, given the inexistence of palpable testicular masses and regular b-hCG value. Normal prolactin levels excluded hyperprolactinemia, and regular levels of hepatic, renal and thyroid function excluded other chronic diseases.

The presence of an autism spectrum disorder caused the consideration of genetic diseases, namely Klinefelter syndrome and myotonic dystrophy type 1. In Klinefelter syndrome, serum-free testosterone levels are low-normal or frankly low, whereas serum estradiol levels are normal or elevated. The resulting increase in the circulating estrogen/androgen ratio leads to gynecomastia.^8^ However, normal karyotype excluded the diagnosis of Klinefelter syndrome.

According to family history, molecular study of the DMPK gene was carried out, confirming the diagnosis of myotonic dystrophy type 1. It or Steinert’s disease is an autosomal, dominantly inherited disorder caused by the expansion of an unstable trinucleotide CTG repeated sequence in the 3’ untranslated region of the DMPK gene on chromosome 19q13.3.^9^ ^,^ ^10^ ^,^ ^11^ ^,^ ^12^ ^,^ ^13^

The myotonic dystrophy type 1 is a multisystemic disease and the second most common form of muscular dystrophy.^14^ Several hypotheses have been proposed to explain the pathogenesis of this multisystemic disorder, which seems to involve the RNA transcribed from the expanded allele.^14^ The pathogenic mechanisms involve protein sequestration, stimulation of signaling pathways, disruption of alternative splicing, mRNA translation and, possibly, mRNA stability, causing a wide clinical spectrum.^14^

Gynecomastia is a known manifestation of myotonic dystrophy type 1, even though it is uncommon (<10%) and rarely a presentation form of the disease.^8^ Usually, it is seen in older male individuals, associated with hypergonadotropic hypogonadism^5^ and in the context of infertility screening. However, in our case report, the boy was at the peripubertal period, with slight increase of FSH, which may mean the beginning of hypothalamic-pituitary-gonadal axis activation, with doseable estradiol, but without testosterone production. This could have led to imbalance in estradiol/testosterone ratio, with subsequent breast enlargement.

Imbalance in estradiol/testosterone ratio, nevertheless, could be also explained by low levels of adrenal androgen production, seen in peripubertal period, which can serve as precursors for peripheral conversion to estrogens.^8^ Onset of gynecomastia of our case report occurred around the adrenarche period (seven to 14 years), favoring this contributing mechanism. The beginning of pubarche reflects the activity of adrenal gland, which leads to the production of androgens. Aromatase is a key player in estrogen synthesis and converts androgen to estrogen.^9^ In case of increase of aromatization activity, androgens are converted into estrogens that cause breast enlargement. In hypothesis, there could have been an increase of aromatization activity resulting from “gains-of-function” of the mutant RNA, causing breast enlargement at a prepubertal age. On follow-up, pubertal development was observed, and gynecomastia remained stable.

Myotonic dystrophy type 1 is a disease with high morbidity, which may be associated with early death (at the fifth decade of life), mainly due to cardiorespiratory failure (70%).^15^ There is currently no cure, but proactive management is likely to significantly reduce morbidity and mortality.^15^

Follow-up includes regular monitoring of cardiac function to screen conduction disturbances and tachyarrhythmias,^15^ ^,^ ^16^ screening of other endocrine dysfunction by regular analytical evaluation, and regular ophthalmologic assessment for cataract detection. At each consultation, it is important to question about muscle symptoms, namely myotonia and muscle weakness, gastrointestinal symptoms (swallowing difficulties, postprandial vomiting/bloating/nausea and weight loss), and bladder dysfunction (incontinence, frequency and urgency).^15^

We presented the case of a prepubertal adolescent with autism spectrum disorder and bilateral gynecomastia, who was diagnosed with myotonic dystrophy type 1 without other symptoms or anomalies in the observation, namely in muscle strength and relaxation. Analytically, there was an increased estradiol/testosterone ratio. As compared to the adult form of disease, endocrinopathy has been rarely documented in childhood myotonic dystrophy type 1 patients,^11^ and we are not aware of another case similar to ours described in the literature.

Prepubertal gynecomastia must ensure the investigation for possible underlying diseases. Myotonic dystrophy type 1 in childhood manifested by autistic disorder and gynecomastia is rare, especially in the absence of muscular symptoms. This case highlights the importance of considering the diagnosis of myotonic dystrophy type 1 in the presence of endocrine and neurodevelopmental manifestations.
